# Learning to live with Parkinson’s disease in the family unit: an interpretative phenomenological analysis of well-being

**DOI:** 10.1007/s11019-016-9716-3

**Published:** 2016-06-30

**Authors:** Laura J. Smith, Rachel L. Shaw

**Affiliations:** 1 Cancer Research UK, London, UK; 2 School of Life and Health Sciences, Aston University, Birmingham, B4 7ET UK

**Keywords:** Parkinson’s disease, Lifeworld, Caregiver, Diagnosis, Disease management, Qualitative research

## Abstract

We investigated family members’ lived experience of Parkinson’s disease (PD) aiming to investigate opportunities for well-being. A lifeworld-led approach to healthcare was adopted. Interpretative phenomenological analysis was used to explore in-depth interviews with people living with PD and their partners. The analysis generated four themes: *It’s more than just an illness* revealed the existential challenge of diagnosis; *Like a bird with a broken wing* emphasizing the need to adapt to increasing immobility through embodied agency; *Being together with PD* exploring the kinship within couples and belonging experienced through support groups; and *Carpe diem!* illuminated the significance of time and fractured future orientation created by diagnosis. Findings were interpreted using an existential-phenomenological theory of well-being. We highlighted how partners shared the impact of PD in their own ontological challenges. Further research with different types of families and in different situations is required to identify services required to facilitate the process of learning to live with PD. Care and support for the family unit needs to provide emotional support to manage threats to identity and agency alongside problem-solving for bodily changes. Adopting a lifeworld-led healthcare approach would increase opportunities for well-being within the PD illness journey.

## Introduction

Parkinson’s disease (PD) is a progressive, debilitating neurological condition usually diagnosed between 50 and 65 affecting 127,000 people in the UK (Parkinson’s UK 2014) and approximately 60,000 in the US (Parkinson’s Disease Federation 2016). Incidence worldwide is greater in men (58.53 per 100,000 aged 60–69) than in women (55.10 per 100,000 aged 60–69) but across both sexes incidence increases with age (104.99 per 100,000 men and 135.72 per 100,000 women aged 70–79) (Hirsch et al. 2016). Degeneration of dopamine-producing cells in the grey brain matter leads to slow movement, resting tremor, rigidity and abnormally diminished muscle movement leading to very little movement altogether. Although PD is not life-threatening, it can be life-altering. Diagnosis presents possibilities for relief at naming the condition responsible for symptoms and anxiety surrounding an unknown future (Phillips 2006).

Previous research has investigated people’s experience of living PD in-depth but it tends to have focused either on the patient or caregiver; here we look at the patients and their partners within the family unit. People living with PD (PwPD) have described diagnosis as ‘dropping the bomb’, signifying its devastation (Phillips 2006). Diagnosis can manifest as lost agency and altered sense of self as PwPD begin to work through this biographic disruption (Bury 1982), making comparisons between their ‘new’ and ‘previous’ self (Bramley and Eatough 2005). Loss of control is maintained in advanced PD through continuously needing to come to terms with an unpredictable body (Haar et al. 2011). Alongside lost agency and lack of bodily control come threats to independence, which serve to strengthen the impact of PD on a person’s lifeworld (Fine and Glendinning 2005; Marcum 2004).

The likely caregiver for PwPD is an elderly spouse (Parkinson’s Disease Society 2008). Like PwPD, partners often felt unprepared and troubled by diagnosis and received minimal support (McLaughlin et al. 2010). The progressive nature of PD can be especially challenging for caregivers as their duties increase (Tan et al. 2012). However, it was the emotional aspects of caring for a spouse that were experienced as most challenging (Tan et al. 2012). Indeed, we know that ‘caregiver burden’ can impact on physical and mental health (Parkinson’s disease Society 2008), thus emphasizing the need to understand living with a degenerative condition at the family level.

Indeed, diagnosis of a long-term illness does not just affect the individual but also the family unit, yet little research investigates illness at the family level (for notable exceptions see: Semple and McCaughan 2013; Burton et al. 2013). To enable an in-depth examination of the lived experience of PD within the family we adopted a lifeworld-led approach to this study (Galvin and Todres 2011). The lifeworld constitutes everything within our horizon of experience; relationships, place, time, opportunities; including those aspects of life which are unique and those that are shared within the world (Ashworth 2006). The lifeworld represents our connectedness with the world, illustrating the importance of understanding life events like illness in a relational sense, in their context (Husserl 1970). As well as this relatedness, Heidegger (1962) emphasized the significance of time within our lived experience—or lifeworld—especially when confronted with life events that change our view of the world and of our future.

Time, reflecting on past and future selves, unsurprisingly features within experiential accounts of living with PD. Heidegger (1962) argued that we are future oriented, making sense of the present in relation to our anticipated future. This prospect of future—mobility—has been identified as fundamental to our well-being (Todres and Galvin 2010). Hence, any disruption to our future expectations results in an ontological challenge. Also significant to our well-being is the concept of being-at-home-with what has been given—dwelling (Todres and Galvin 2010). Galvin and Todres (2011) bring these two concepts from Heidegger together, articulated within the ‘dwelling-mobility lattice’ of well-being (see Table 1). Mobility is the feeling of possibility, living towards the future; dwelling is a feeling of acceptance within a situation, grounded in the present (Todres and Galvin 2010). Dwelling-mobility encompasses the freedom of mobility and peace of dwelling: “a feeling of rootedness and flow, peace and possibility” (Galvin and Todres 2011: 65). To understand family members’ experience of living with PD, we investigated their lifeworlds to identify how opportunities for well-being played out differently and how they were impacted by different dimensions of the lifeworld.

**Table 1:** ‘Dwelling-mobility lattice’ adapted from Galvin and Todres (2011) theory of well-being used to help make sense of the data

Element of the lifeworld	Mobility	Dwelling	Dwelling-mobility
Spatiality	Adventurous horizons	At homeness	Abiding expanse
	Anything that offers a place of promise. A sense of adventure where spatial possibilities arise that offer movement (metaphorically or literally)	A sense of being at-home, feeling of being settled/still within the physical environment. Familiar and comfortable surroundings or having familiar/personal objects close-by	Both a feeling of at-homeness with possibilities for adventurous horizons. Being deeply connected to a place but also opportunity to go further afield (metaphorically or literally)
Temporality	Future orientation	Present-centredness	Renewal
	Being energised by future possibilities (metaphorical or literal) which emphasize the sense of flow, not being stuck	Absorbed in the present in a way that is desired. Sense of belonging, being ‘in the zone’	Unification of future possibilities with a satisfaction with the now. Rooted flow—a sense of being absorbed in the now and a welcome readiness for the future
Intersubjectivity	Mysterious interpersonal attraction	Kinship and belonging	Mutual complementarity
	In tune with interactional possibilities and an attraction to people’s ‘otherness’, understanding the mystery of others	An effortless being together; ‘we’ rather than ‘I’ and ‘you’	Both a sense of kinship/togetherness and excitement at learning new things—a ‘homelike oneness’ and difference
Mood	Excitement or desire	Peacefulness	Mirror-like multidimensional fullness
	Sense of ‘attunement’ and buoyancy of movement (looking forward to a longed-for holiday or special event)	Stillness, settledness. There is a welcomed pause, coming to accept things, and ‘letting be’	Complex mood encapsulating an energy of moving forwards and a sense of being at one with the world and oneself
Identity	‘I can’	‘I am’	Layered continuity
	Sense of being able to. Experiencing oneself as being on the move (literally or metaphorically)	A sense of self that is supported by continuous histories and contexts that fit with who ‘I am’	A continuous sense of ‘I can’ and a strong sense of ‘just being’ in a foundational sense. Ontological security
Embodiment	Vitality	Comfort	Grounded vibrancy
	Tuned into an embodied energy that offers the possibility of movement, ‘bodying forth’	Literal feeling of comfort, warmth, relaxation. Felt sense of familiarity and intimacy with one’s body	An energized flow and a bodily sense of feeling deeply at-home. Both ‘being’ and ‘becoming’ is possible

The biographical disruption (Bury 1982) experienced in and following PD diagnosis resonates with Heidegger’s notion of homelessness. When we are well, we feel at-home in our bodies, but when illness strikes we begin to feel “disharmony, dis-equilibrium, dis-ability, and dis-ease which incorporates a loss of the familiar world” (Toombs 1993: 96); a sense of existential homelessness. We have seen that adapting to bodily changes can have a significant impact on an individual’s identity and relationships. Nevertheless, Heidegger’s theory proposes that facing up to these challenges and coming through the other side can be experienced as well-being, a kind of homecoming (Dahlberg et al. 2009). An authentic homecoming is represented by the co-existent peace of feeling at-home (dwelling) and possibilities for moving forward (mobility)—dwelling-mobility.

The summary above demonstrates the existence of high quality qualitative research in this area focusing either on the patients’ or caregivers’ experiences. Instead of separating out these accounts, we look at them together as voices representing the family unit. Furthermore, we investigate partners’ accounts in their own right, that is, not in their role of carer, but as another equally valid perspective of the experience of living with PD. Taking this idiographic approach to both patients’ and partners’ experiences of living with PD required a methodology committed to making sense of subjective experience within the relational realm, that is, within the relational world of the family but also alongside existing relationships in the world.

To achieve this, we adopted a phenomenological approach drawing on theoretical insights from Heidegger’s (1962) notion of homecoming and Todres and Gavlin’s (2010) theory of well-being, which incorporates the concept of dwelling-mobility. The theoretical roots of the study are within phenomenology and drive the research question: what is family members’ lived experience of PD and what are their opportunities for well-being?

A secondary objective of this work was to examine the role played by theory in a phenomenological analysis. We adopted interpretative phenomenological analysis (IPA; Smith et al. 2009) as the method of analysis and stayed true to its idiographic commitment and prioritization of understanding individuals’ meaning-making processes of ontologically challenging experiences. Accounts were analysed inductively following a ‘typical’ IPA approach (see Smith et al. 2009). The themes generated were then considered alongside Todres and Galvin’s (2010) theory of well-being through the process of abductive reasoning (Hiles 2014).

## Methods

### Design

This study adopted a phenomenological approach which prioritized participants’ lived experience of PD. It was framed within an explanation-driven logic of inquiry (Hiles 2014) involving abductive reasoning, i.e. inference to the best explanation (Harman 1965). Individual accounts were analyzed using IPA and themes were then interpreted in consultation with Galvin and Todres (2011) existential-phenomenological theory of well-being.

### Participants

Ethical approval was granted by the University Ethics Committee to recruit PwPD and their partners via a Parkinson’s UK support group in the midlands of England.

### Data collection

Individual in-depth interviews were conducted in participants’ homes, which were audio-recorded and transcribed verbatim. Open-ended questions focused on diagnosis and living with PD, including, e.g.: ‘Can you describe your feelings when you were diagnosed with Parkinson’s disease?’ ‘Tell me about a typical day, living with PD.’ ‘As a partner of someone living with PD, has your life changed?’

### Data analysis

Data were analysed using interpretative phenomenological analysis (IPA; Smith et al. 2009). Analysis was true to the idiographic commitment of IPA; each case was analysed and initial themes identified before moving on to the next case. Coding involved looking for patterns in language use, metaphors/imagery, and identifying the concerns of participants as PwPD and their partners. Themes for each case study were then clustered to establish an in-depth, experience-close description of learning to live with PD.

Galvin and Todres (2011) proposed that well-being is an experiential phenomenon intertwined with different elements of the lifeworld. As such, it can hold multiple characteristics simultaneously. In this analysis, we used Galvin and Todres’ (2011) ‘dwelling-mobility lattice’ to help discern the nature of participants’ well-being and how it was affected in different ways by different dimensions of the lifeworld, i.e. temporality, spatiality, intersubjectivity, mood, identity, and embodiment (see Table 1). This meant that following the inductive IPA analysis, a further analysis was undertaken using abductive reasoning to consider themes generated alongside the existing theoretical concepts within Galvin and Todres’ theory of well-being, summarised in the dwelling-mobility lattice. This final process helped ground the inductive analysis of the phenomenon of interest—the lived experience of PD—within phenomenological theory about the quality of well-being when living with illness.

## Results

Nine participants were interviewed (see Table 2 for details): four PwPD and their partners—Richard and Susan, Jeremy and Lesley, Ann and Roger, Helen and George, and Jane whose partner, Albert, who had PD, had died 2 years previously. Themes are presented in turn (see Table 3 for a brief summary).

**Table 2:** Demographic details of participants

Pseudonym	Gender	Age	Patient/Partner	Years since diagnosis
Richard	M	67	Patient	2
Susan	F	67	Partner	
Jeremy	M	75	Patient	21
Lesley	F	70	Partner	
Ann	F	71	Patient	21
Roger	M	74	Partner	
Helen	F	75	Patient	21
George	M	85	Partner	
Jane	F	74	Partner (partner deceased)	15

**Table 3:** Themes generated in the analysis with notes about their content

Theme	Notes
It’s more than just an illness	Myths about PD
	Diagnosis as a bombshell
	Disruption of self
	Ontological challenge
	Being taken over by PD
Like a bird with a broken wing	Damaged/broken
	Adapting to bodily changes
	Self-reparatory actions
	Embodying agency
Being together with PD	Kinship with partnership
	Connectedness
	Sense of duty
Carpe diem!	Uncertain future
	Making the most of now
	Attempt to break the future orientation

### It’s more than just an illness

This theme explores the meanings attributed to PD by participants. Ann and Jane both tapped into ‘mythical’ or stereotypical images of PwPD as, respectively, “*a funny old man in the corner of the pub dribbling*” and “*an old person that shakes*”. But Jane went on to say how this did not do justice to the person at the centre of this condition.

…most people’s perception of Parkinson’s is an old person that shakes, erm, that is the average perception of it, not, here is still an intelligent person, who unfortunately has got some neurological disease, which is attacking their brain (Jane, partner)

Jane’s clarification positions the PwPD as a victim to a bodily attack. Pre-diagnosis, that assault on the body was experienced as subtle, even inconsequential. Participants’ knowledge of physical symptoms of PD was limited meaning that a diagnosis was often experienced as a shock.

…well a bit of a bombshell really but I think he had symptoms, which we wouldn’t even have dreamt were Parkinson’s symptoms, erm, knowing very little about it (Susan, partner)

This “bombshell” resonates with Phillips’ (2006) work and further illustrates the disruptive and life-changing nature of PD diagnosis. With the degenerative character of PD came mystery and uncertainty, which when coupled with a lack of knowledge created a powerful ontological challenge for PwPD and their partners.

I knew that I’d read it was long term, but I couldn’t absorb that, er and really accept it and I had a close member of family who’d been an auxiliary nurse, who knew exactly what it would lead to who didn’t tell me, but I could tell from their facial expressions when talking about Parkinson’s er that they, it, there was something more to it than just an illness that would go away. (Lesley, partner)

Lesley’s phrase, *“more than just an illness”*, indicated how a PD diagnosis meant more than being ill in an ordinary way; it meant learning to live in a new way. Ann described how she felt that she had been *“taken over”* by PD in that it dictated what she was able and unable to do.

…if I didn’t have him to answer the phone and organise, I’d have never have been able to re-organise this morning like we did with having the diabetic nurse first and a quick 5 min for my breakfast and them erm fluid and that, and he, he just liaises, he even buys the birthday cards now, we, which was everything I used to do, in fact he’s taken over me diary, I feel taken over (Ann, patient)

Initially, Ann seemed pleased that Roger was able to take care of things for her. However toward the end of the extract, for Ann, there is a sense of being *“taken over”* by PD *and* her husband; PD has limited her capacity to do what she used to do and Roger has taken up those tasks. The job Ann mentioned specifically, buying birthday cards, may appear mundane but could easily be filled with feelings of joy and love when buying gifts for family and friends; this is a loss of more than running an errand.

These extracts have illustrated the powerful and disruptive effect a PD diagnosis can have in relation to a person’s identity, a feeling of ‘I can’ being replaced with ‘I can’t’; it also throws up obstacles to our future orientation in that time becomes fragile as uncertainty of the nature of degeneration slowly becomes a reality.

### Like a bird with a broken wing

As we age, we expect things to start breaking. George said as much: *“well we’re both getting old so you expect things to happen”*. It is not uncommon for people to rationalize long-term conditions, like PD, simply as part of ‘normal’ ageing. For Susan, living with Richard as a PwPD was far more difficult than that.

I think, my understanding of it now and living with it day to day, it’s not, it’s like anything, it’s like the unknown, it, that’s the frightening bit what we’re living through is just our lives, it’s not, this isn’t the nightmare, it’s just our lives with that thrown in, if you see what I mean (Susan, partner)

The *“nightmare”* of living with PD threw up constant challenges for Susan which seemed to present a kind of ‘inauthentic homelikeness’; in one way it sounds like Susan is trying to normalize the challenges embedded within her experience with Richard but there is a sense of her not being okay with it. Indeed, that is corroborated by Richard: *“I mean something that would take me an hour, might take me three now to the fact that I don’t attempt it anymore.”* Later on, though, Richard is more optimistic: *“as long as I can do it, I will do it”*. In Ann’s account also there is a feeling of hope despite the daily struggles she experiences.

…it’s like, an animal who has a wing broken, a bird who’s got a broken wing, it will still feed and move around, even though it’s holding the wing and it’s the same with patients with Parkinson’s (Ann, patient)

This powerful metaphor symbolizes Ann’s lost freedom as she was no longer able to fly away; opportunities for mobility (literally and figuratively) had gone. Nevertheless, by holding her broken wing, she could find ways of supporting herself and adapted to her newfound circumstances. George also spoke about Helen’s ability to adapt with the help of supportive devices.

Well you just take it as it comes and you change your routine, as you need to. I mean we, we, find we’ve got three walking devices now, we’ve got a Zimmer frame, we’ve got a four-wheel walker that she used to use and now we’ve got a wheelchair (George, partner)

Using devices in this way enabled Helen and George to retain a sense of vitality and thereby opportunities to maintain involvement in activities outside the home. George and Helen’s use of devices and Ann’s insistence to *“float back up again”*, even with a broken wing, illustrates the importance of agency in coming to terms with PD and taking action to overcome the barriers it presents.

### Being together with Parkinson’s disease

This theme explores the relationship of the couples interviewed and their connections to others. Richard quite clearly said that his diagnosis had had a positive impact on his relationship with Susan, making them realise how much they loved each other: *“I mean we sort of found out our feelings for each other have sort of multiplied. We’ve got a lot more time for each other”*. This kinship and belonging was a source of well-being for Richard. Ann focused on the practicalities, the interdependence, of her partnership with Roger now she was living with PD: *“the two of us make up one whole well*-*equipped person”*. Roger also spoke of his and Ann’s togetherness, but for him there was an encroaching feeling of frustration, which threatened his autonomy.

…she can’t really do anything for herself now, so everything she wants to do, I have to do, and the same as if I wanna do something at the same time, then you know er sometimes I can lose my temper (Roger, partner)

Jane echoed this sense of frustration at the need to provide constant care, because she couldn’t leave her husband alone. But Jane was able to translate it from a potential loss of autonomy into an opportunity for enjoyable togetherness: “*I didn’t have a life of my own, but we did things together because I made it that way otherwise, well, you wouldn’t go out would you?”*. Jane was able to integrate Albert’s need for her to be there with her own need for social engagement. Helen described her engagement with the PD support group as a source of companionship and belonging.

…they’re a great crowd, yes, there’s less of us now, erm but there’s new people coming in, but some don’t want to come, some don’t want to join in but, we go and have a coffee and a chat about and we have, we go out for lunch occasionally and we have lectures, I mean last month there was a solicitor there erm sorting out how to do you wills and so forth and how you save your tax (Helen, patient)

Helen’s account acknowledged others’ reticence to *“joining in”* support groups but she also stressed that they do not only offer opportunities for social interaction but also information which can enable PwPD to maintain control over their financial affairs, thus retain a sense of ‘I can’ in amongst the difficulties. Use of support as an ‘optimizing strategy’ (Baltes 1997) here is similar to Helen and George’s use of mobility aids; they seem to have come through the other side and been able to assimilate PD within their lives.

### Carpe diem!

This theme describes changes in participants’ perceptions of time. The PD diagnosis had created a sense of urgency in people’s lives.

… I think I was aware that you, whilst you’re healthy, you’ve got to do things erm, and not procrastinate and put them off, do what you can now, erm I mean not that we’ve gone round the world or anything like that but if there’s an event on or, say “Come on let’s go” rather than “Oh, we can go next year”. (Lesley, partner)

This represents a feeling of temporal renewal for Lesley and Jeremy; opportunities which may have been postponed or not contemplated were now taken up in the present and near future. Lesley’s tone betrays an uncertainty (“*whilst you’re healthy”*) that comes with the degenerative nature of PD. She elaborated this point, revealing the significance of this uncertainty to her understanding of the future.

…And with Parkinson’s you seem to plateau, you know there’s a slight deterioration and then a plateau for a few months, so you, you can relax and then you realise there’s another slight problem so we deal with that and then cope for a few more months so it’s a gradual process. (Lesley, partner)

This gradual decline seems to impede Lesley’s ability to fully relax and let be. This fracture in Lesley’s expectations of her future life has maintained an ‘unhomelikeness’ which has prevented her thus far from fully coming to terms with PD. Ann also expressed a reticence at looking too far ahead.

…I haven’t thought that far, I don’t wish, I think in some ways I don’t wish to feel that far, because I, you know, forever be in a state of change, because if something happened to Roger, it would not only be Roger it would be both of us, it would be devastation really (Ann, patient)

This extract, like others above, underlines Ann’s dependence on Roger. It also signifies her attempt to break away from her future orientation but the words she used centre on her mood, her feelings: *“I don’t wish to feel that far”*. Imagining the future in terms of her body is one thing; imagining how she will feel about her identity, Roger, her life, is even more difficult. Like Lesley, it appears that Ann may still be working through her ontological challenge. For Susan, there is light at the end of the tunnel after some taxing times for her and Jeremy.

We do what we wanna do now, you know, we’ve had a bit of a tough few years so yeah, we’re making it our time now (Susan, partner)

There is a sense of having come out the other side here and a glimpse of the homecoming Heidegger described: *“we’re making it our time now”* has a sense of renewal in Susan’s perception of the present and the future but also a sense of ontological security in who she is as partner to Richard, living with PD. They are safe and able to live with agency.

This analysis has revealed the complexity involved in living with PD for the PwPD and their partners. Limited knowledge about PD meant their journeys began in the unknown. Fear of this unknown was exaggerated by the uncertain future and bodily decline. These changes worked to threaten participants’ identities and fractured their perceptions of time. Some were still working through these challenges while others seemed to have experienced something akin to an authentic homecoming.

## Discussion

This research explored the lived experience of living with PD from the perspectives of PwPD and their partners. Framing the study within a lifeworld-led approach using IPA enabled an idiographic analysis which highlighted the experiential journey of learning to live with a degenerative disease within the family. Ageing—complete with chronic conditions and increasing immobility—has been described as a “fundamental ontological challenge” (Gilleard and Higgs 2010: 125); this was certainly the case for individuals interviewed here. Adjusting to reduced bodily capacity and the implications that has on identity were experienced as an emotional struggle by partners as well as PwPD, supporting Bramley and Eatough (2005). Adapting to life with PD also involved bodily challenges. Indeed, Berglund and Källerwald (2012) found that diagnosis with a long-term condition altered individuals’ body and therefore access to the world. Hence, to come to terms with PD, Berglund and Källerwald (2012) argued that people needed to experience their illness as an embodied process for them to move toward a new self-understanding.

Participants who had made adjustments were able to assimilate PD into their lives and retained their agency. Following Berglund and Källerwald (2012) proposal, by allowing PD into their lives, some participants had genuinely come to understand it as a part of their lifeworld. This authentic homecoming facilitated a resource-oriented approach to living with PD, as demonstrated by those using mobility devices and adaptations to daily activities, which offered opportunities for social engagement while maintaining the ability to provide care. Using care and support as an ‘optimizing strategy’ (Baltes 1997) thus enabled participants to make the most of their situation instead of getting overwhelmed by their disabilities and deficits.

However, as Haar et al. (2011) found, living with PD is a fluid experience with ever-increasing threats to agency, control and independence. Living through this existential struggle emphasizes the need to realise the co-existence of well-being and suffering. Thus, learning to live with a degenerative condition in later life needs to be understood in terms of “the potentials *and* limitations, the pleasures *and* sufferings, the continuing vitality, competence *and* vulnerability” (Baars and Phillipson 2013: 26). Attempting to understand the lifeworlds of families living with PD offers possibilities for greater understanding of their vulnerabilities and subsequently opportunities to provide tailored support that will make a difference to their lives. We know that coping is a dynamic process that shifts around in response to the objective demands and subjective appraisal of a situation, i.e. the need for mobility devices alongside the need for kinship and belonging (Folkman and Lazarus 1985). Previous research identified active problem-solving and acceptance as most effective in reducing anxiety and associated with better health outcomes (Penley et al. 2002). However, these findings support others who have emphasized the need for emotional support alongside that problem-solving when coming to accept PD as part of the lifeworld (Berglund and Källerwald 2012; Dahlberg et al. 2009). That sense-making involves coming to terms with being-with-what-has-been-given (dwelling), thus, enjoying the well-being of being at-home with oneself, one’s body, and one’s being-in-the-world (Galvin and Todres 2011).

The fluidity of PD and its progressive nature led to changes in participants’ perception of time. Berglund (2014) also found that living with long-term conditions led to a need to take advantage of the time left by living in-the-moment. Heidegger (1962) expressed this as being-towards-death, i.e. our future orientation intimately links our being-in-the-world with the prospect of death. For Heidegger, relating to a finite temporality represents a key difference between an authentic or inauthentic relationship to death; an authentic grasp of death allows us full appreciation of life. In other words, living with a long-term condition like PD changes our horizons which can lead to a greater sense of appreciation of the present, experienced as well-being (Carel 2006).

Participants were recruited through Parkinson’s UK support groups, and the partners gained as much from it as the PwPD. Indeed, Charton and Barrow (2002) found that members of support groups were better equipped to accept PD and to make adjustments to their lives. By comparison, non-members were more likely to deny the condition a central part in their lives (Charton and Barrow 2002). This study has explored the experience of living with PD from the perspective of the PwPD and their partner. It has shown that the challenges faced by PwPD are echoed in their partners’ experiences as they go through a shared journey but with different emphases. The PwPD needed to adjust to an altered sense of self established over time following bodily changes, something which is catered for in support groups; their partners were learning to become caregivers whilst attempting to retain a sense of autonomy in the life choices they made, something which perhaps is missing in support offered. This supported McLaughlin et al.’s (2010) finding that as PD progressed there was an increasing reliance on caregivers, which came with an emotional and social burden. This also corroborates previous suggestions that more support is needed for caregivers (Tan et al. 2012). We know that PwPD living with partners are more likely than those living alone to experience higher levels of well-being (Cubí-Mollá et al. 2014). This research has illustrated the shared nature of the illness journey which points towards a need for services designed for the family unit which could benefit the caregiver as much as the PwPD.

Through an in-depth examination of the lived experience of PD within the family, this study has illustrated the existential impact of diagnosis on both the PwPD and their partner. By seeking to understand the families’ lifeworlds we have illuminated the need for recognition of this impact on different elements of the lifeworld, particularly identity and its intimate links to embodiment and temporality. We have also found that adapting to a life with PD requires pro-active sense-making and adjustments to assimilate PD into everyday life; taking control by facing up to the challenges of PD makes way for a more positive mood and therefore a better sense of well-being (dwelling-mobility). The need for a feeling of belonging was important to PwPD and their partners, some of which was satisfied by being-together-with PD, some was achieved through social engagement and companionship through support groups. Learning to live with an uncertain future was perhaps most challenging because it requires us to face our own mortality and break the pattern of making sense of life through our expectations of the future. Seizing the day (carpe diem!) helped some couples to appreciate the present and achieve well-being.

More research is needed to examine the experience of the family further, including adult children and wider family members. In particular, further work is required in extended families to understand the lifeworlds of older adults living with adult children or other family members when diagnosed with PD. In contrast, the experience of older adults living alone in the community or in assisted living environments or retirement communities would provide a better understanding of the lived experience of PD to help identify the range of services needed for those living in different types of households with different opportunities for companionship and support. Future research also needs to investigate PwPD and family members who do not to access support facilities available to identify their reasons for non-engagement and to better understand how we might enhance their well-being through access to services that meet their needs.

Taking a phenomenological approach in this research within an explanation-driven logic involving abductive reasoning, i.e. creating a dialogue between data generated and existing theoretical constructs, enabled us to make recommendations for lifeworld-led healthcare based on our own empirical work and that of others used to develop those theories (Todres et al. 2007). Healthcare that takes into account the family context for the PwPD but also the existential challenge it throws up for both the PwPD and their partner. For both parties to experience well-being, support needs to be available which can help them to pro-actively come to terms with what the diagnosis means *for them* in order for them to accept it (feel at-home with their newfound reality—dwelling) and to make necessary adjustments to their lifestyle in order to maintain opportunities for the future (mobility). Ideally, we need to help PwPD and their partners to work through the challenges together towards a genuine acceptance—an authentic homecoming—offering optimum well-being in the circumstances in which they find themselves (dwelling-mobility) (Todres and Galvin 2010). In short, a lifeworld-led approach to healthcare would re-humanize services provided and help the PwPD and their families to work through the ontological challenge of living with PD and offer them a smoother pathway along their illness journey.
